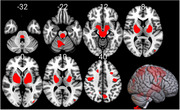# The global tau severity network in progressive supranuclear palsy

**DOI:** 10.1002/alz70856_100644

**Published:** 2025-12-25

**Authors:** Chiung‐Chih Chang

**Affiliations:** ^1^ Kaohsiung chang Gung Memorial Hospital, Kaohsiung, NA, Taiwan

## Abstract

**Background:**

The second‐generation tau tracer, Florzolotau (18F) PET, has demonstrated high specificity in diagnosing progressive supranuclear palsy (PSP). Developing a standardized quantification method to reflect global tau burden is crucial for predicting cognitive and motor severity. This study aimed to establish a global tau severity (gTS) score using Florzolotau (18F) PET in PSP patients.

**Material and Methods:**

This study was conducted at two teaching hospitals, Chang Gung Memorial Hospital Kaohsiung and Linkou campuses, and included a pilot cohort of 15 cognitively unimpaired age‐matched controls (CTL) and 15 PSP patients, followed by a validation cohort of 94 CTL and 116 PSP patients. In the pilot cohort, we developed a PSP‐specific tau mask and identified the optimal reference region for Florzolotau (18F) using effect size analysis. In the validation cohort, we determined the gTS score cutoff value for group stratification and assessed its correlation with cognitive (MMSE), motor (UPDRS), and disease severity (Progressive Supranuclear Palsy Rating Scale, PSPrs) measurements.

**Results:**

A gTS cutoff value of 35.9 for PSP achieved the highest area under the curve (AUC = 0.901) with a specificity of 0.92 and sensitivity of 0.75 (Youden's index = 1.665). Regression analysis revealed significant correlations between the gTS score and cognitive (MMSE, ρ = ‐0.26, *p* =  0.005), motor (UPDRS, ρ = 0.269, *p* =  0.006), and disease severity (PSP Rating Scale, r = 0.372, *p* =  0.0001) scores.

**Conclusions:**

The network score provides a reliable measure of tau burden and is significantly associated with cognitive and motor severity in PSP. This standardized metric offers potential for clinical application in assessing disease progression and stratifying patients based on tau burden.